# A Prospective Cohort Study of Primary Dengue Virus Infection in Medellín, Colombia

**DOI:** 10.3390/vaccines13070748

**Published:** 2025-07-12

**Authors:** Andrea Trujillo, Liesbeth Van Wesenbeeck, Lina Salazar, Liliana López, Lotke Tambuyzer, Annemie Buelens, Kim De Clerck, Oliver Lenz, Leen Vijgen, Marnix Van Loock, Guillermo Herrera-Taracena, Iván Darío Vélez, Freya Rasschaert

**Affiliations:** 1 Grupo de Investigación Clínica-PECET (GIC-PECET), Universidad de Antioquia, Medellín 050010, AN, Colombia; andretru@gmail.com (A.T.); linasalazar@pecet-colombia.org (L.S.); liliana.lopez@pecet-colombia.org (L.L.); 2 Johnson & Johnson, 2340 Beerse, Belgium; lvwesenb@its.jnj.com (L.V.W.); lotketambuyzer@gmail.com (L.T.); abuelens@its.jnj.com (A.B.); kdecler1@its.jnj.com (K.D.C.); olenz1@gmx.net (O.L.); lvijgen@its.jnj.com (L.V.); mvloock@its.jnj.com (M.V.L.); 3 Johnson & Johnson, Horsham, PA 19044, USA; gherrer8@its.jnj.com; 4 Programa de Estudio y Control de Enfermedades Tropicales PECET, Universidad de Antioquia, Medellín 050010, AN, Colombia; ivan.velez@udea.edu.co

**Keywords:** dengue virus, dengue naïve, traveler’s population, seroconversion, Colombia

## Abstract

Background: The evaluation of antiviral or vaccination strategies for the prevention of dengue infections in a traveler population would require extensive and complex studies. This prospective study aimed to identify a cohort of dengue naïve participants living in Medellín, a dengue endemic area, as a proxy for travelers and to determine the incidence of primary dengue virus (DENV) infection (symptomatic and asymptomatic) in this cohort. In Colombia, epidemic dengue waves occur every 3–4 years, with infected *Aedes* mosquitoes present in ~80% of the territory, including Medellín. Methods: Participants > 16 years of age, living in Medellín, were screened for anti-DENV immunoglobulin G (IgG). DENV seronegative participants were enrolled in this study. A serological anti-DENV survey was performed, with semiannual sample collections for up to 2 years. Acute DENV infections were evaluated by monitoring fever and testing for DENV nonstructural protein 1 and/or RNA. Results: Of the 4885 screened participants, 3008 participants (62%) were DENV seronegative and enrolled. Among them, 2263 (75%) completed this study, and 2644 (88%) had at least one serosurvey visit after baseline. Of those, 52 (2%) had laboratory-confirmed DENV seroconversion, and 19 (<1%) had febrile illness, but none had laboratory-confirmed DENV infection. Conclusions: This study identified a cohort of predominantly students, seronegative at study start, living in Medellín and serving as a proxy for a prospective DENV infection traveler population. Laboratory-confirmed primary DENV infection was found in 2% of participants, with <1% reporting febrile illnesses, meeting the WHO criteria for probable clinical dengue cases.

## 1. Introduction

Dengue is one of the most common vector-borne diseases worldwide and is caused by infection with one of the four dengue virus serotypes (DENV-1 to DENV-4). Dengue viruses are transmitted to humans by mosquitoes of the *Aedes* species. Colombia is considered a dengue-endemic country with epidemic outbreaks and reported annual incidence rates in the past ten years ranging between 52 cases per 100,000 inhabitants (in 2017) and 273 cases per 100,000 inhabitants (in the epidemic year 2013), and an incidence of 253 cases per 100,000 inhabitants in the most recent full year 2023 [1]. The *Aedes* vector and the four DENV serotypes can be found in approximately 80% of the national territory [2], with regional differences in the epidemiological behavior of dengue during the past reported outbreaks [3]. Medellín is the second-largest city in Colombia, with a population of approximately 2.5 million people. As one of the main urban centers in the country, the city of Medellín attracts many people for access to job opportunities, education, tourism, and other factors. The circulation of the 4 DENV serotypes has been demonstrated within the city and frequently leads to dengue outbreaks [4]. Climate change significantly influences DENV transmission dynamics by altering temperature and precipitation patterns, which expand the habitat range of *Aedes* mosquitoes and increase the frequency of dengue outbreaks, also in previously unaffected regions [5].

Most DENV infections occur without any symptoms. Around 25% of people infected with DENV develop symptoms that include fever in most cases, together with one or more symptoms such as headache, body aches, nausea, or rash [6,7]. Symptoms are generally mild, but a minority of infections can progress to severe dengue disease, which requires hospitalization and can lead to death. Severe dengue disease can occur in both primary and post-primary infections, but in endemic areas it is more strongly associated with post-primary infections, probably due to antibody-dependent enhancement of disease when infection occurs with a different serotype than the primary infection [8].

Laboratory confirmation of dengue cases can be performed by virus isolation, DENV RNA detection using molecular tests, detection of DENV nonstructural protein 1 (NS1), anti-DENV antibody seroconversion, or increases in anti-DENV immunoglobulin (Ig)M or IgG antibody titers, or a combination of these [9]. In Colombia, national guidelines recommend the use of dengue diagnostic tests for surveillance purposes only [10,11]. Most of the case reporting in Colombia is based on a clinical diagnosis of dengue, without requiring confirmatory laboratory testing. Laboratory diagnosis of dengue is not considered necessary for clinical management, except in severe cases or death [10]. For individuals with symptomatic DENV infection, DENV RNA and DENV NS1 antigen can usually be detected during the first 7 days of illness [12]. In the case of primary dengue infection, anti-DENV IgM antibodies generally become detectable 4–5 days after the onset of symptoms, while anti-DENV IgG antibodies appear after IgM, within Day 7 to Day 14 of illness, and can be detected for many months and even years after infection. In post-primary DENV infections, a rapid increase in anti-DENV IgG titers is observed while the IgM response is low or even absent. As DENV serology testing can show cross-reactivity with antibodies against other flaviviruses, results may be difficult to interpret and may require further confirmatory testing [12].

To date, there is no dengue-specific antiviral treatment available, although a limited number of direct-acting antiviral strategies for treatment or prevention are currently in clinical development, including Johnson & Johnson’s mosnodenvir (Phase II), Novartis’ EYU688/NITD-688 (Phase II) and Visterra’s monoclonal antibody VIS513 (Phase II), currently licensed to the Serum Institute of India Pvt. Ltd. [13,14,15]. Two dengue vaccines—Dengvaxia^®^ (CYD-TDV) and Qdenga^®^ (TAK-003)—have been licensed, and a third, the Butantan-Dengue Vaccine, is in late-stage clinical development [16]. However, in 2024, Sanofi Pasteur announced that the manufacturing of Dengvaxia^®^ will be discontinued [17]. The use of Qdenga^®^ is recommended by the WHO in settings with high dengue transmission intensity. Until the efficacy–risk profile for DENV-3 and DENV-4 in seronegative persons has been more thoroughly assessed, WHO does not recommend the programmatic use of Qdenga^®^ in low to moderate dengue transmission settings [18]. Qdenga^®^ was approved in 2023 and has been marketed in Colombia since November 2024 [19]. However, the vaccine is currently only accessible in the private sector and not yet included in the national vaccination schedule in the country.

Travelers from non-endemic dengue areas to endemic-dengue areas are an important population that may benefit from a prophylactic dengue antiviral. However, travelers are highly mobile, and their follow-up once in an endemic-dengue area is logistically difficult to implement. For this reason, the objective of this study was to investigate the incidence of DENV infections in a population of DENV-naïve participants residing in dengue-endemic areas as a proxy for a traveler’s population. The city of Medellín was selected as it is one of the main urban centers in Colombia, with geographical characteristics, covering areas from 1300 to 1750 m above sea level and a temperature between 18 and 26 °C during the rainy season, May to October each year. These conditions are favorable for seasonal dengue outbreaks and the establishment of permanent *Aedes* mosquito vector populations at altitudes below 1600 m. Therefore, recruiting a cohort of young participants living in the outskirts of Medellin at elevations of 1600 m or above but moving to the city center to study or work was taken as an approach to a proxy traveler’s population. Using semiannual serological surveys for the detection of anti-DENV IgG antibodies, the real burden of dengue, including asymptomatic infections, in this population was studied. With this prospective study, we aimed to assess the feasibility of identifying a sizable population to evaluate DENV antivirals or vaccines in clinical trials. This article is a revised and expanded version of the presentation entitled “Dengue virus serologic prevalence and prospective cohort in adult population from the metropolitan area of Medellín, Colombia 2018–2022”, which was presented at 7th Pan American Dengue Research Network Meeting, Pandengue 2023, Lima, Peru, 13–16 November 2023 [20].

## 2. Materials and Methods

### 2.1. Study Design

**Figure 1:**
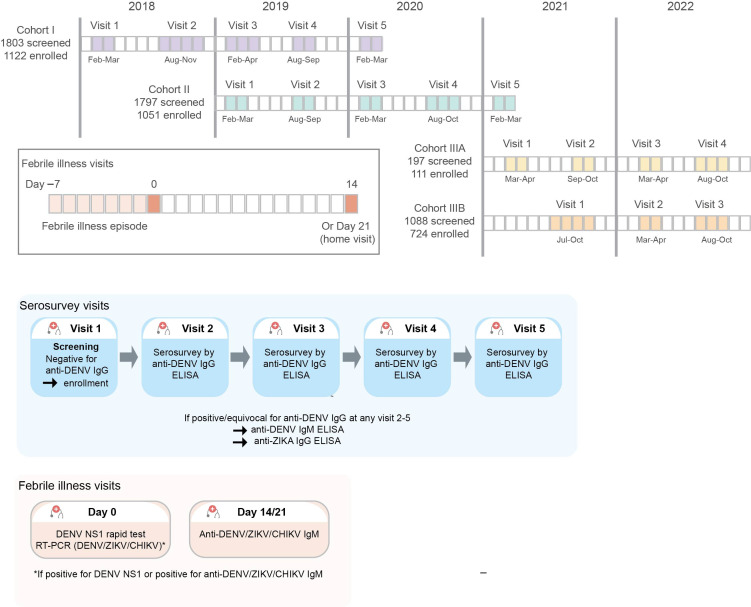
Study design with indication in respective colors of sampling/testing visits.

The prospective cohort study consisted of three cohorts. Cohorts I and II were recruited during the first quarter of 2018 and 2019, respectively, and participants were followed up for 24 months (Figure 1). A third cohort was added, subsequently extending this study by 1.5 years. The initial target population of this study was students from higher-education institutions (universities or technical schools) who had started their academic activities within the last two years. However, since the coronavirus disease-2019 (COVID-19) pandemic and the associated country's confinement policies coincided with the enrollment of Cohort III, the inclusion strategy for Cohort III had to be adapted. Cohort III was therefore recruited from partner companies associated with PECET (Programa de Estudio y Control de Enfermedades Tropicales), in addition to educational institutions. For the educational institutions, the recruitment was carried out through home visits and included not only students but also their family members. Employees of the partner companies were recruited at the workplace. Cohort III was subdivided into Cohort IIIA (recruitment during the first and second quarter of 2021 with 18 months follow-up) and Cohort IIIB (recruitment during the third and fourth quarter of 2021 with 12 months follow-up) (Figure 1). Enrolled participants were followed up with serosurvey visits every six months. The incidence of symptomatic DENV infections was determined by active surveillance; participants were requested (1) to measure their body temperature whenever they felt ill or feverish and (2) in case of fever (≥38 °C) or a history of fever in the preceding 7 days or less to visit the University of Antioquia clinics for clinical evaluation and blood sample collection.

### 2.2. Participants and Study Eligibility

Participants over 16 years of age residing in the city of Medellín and nearby municipalities during the study period, with a negative dengue IgG test (Panbio Dengue IgG Indirect Enzyme-Linked Immunosorbent Assay [ELISA]), who voluntarily expressed their desire to participate and signed the informed consent form, were included in this study.

### 2.3. Recruitment Tools and Retention Strategies

Recruitment strategies focused on the health benefits of participating in this study. These health benefits included DENV diagnostic tests for febrile patients, specialized medical assistance, and health and disease education, such as education on DENV transmission routes, symptom identification, and methods of prevention. At the time of the initial blood sampling, the study participants were provided with a digital thermometer and were instructed to measure their temperature if they felt sick or feverish and to contact designated study staff to report the episode. To enhance study retention, active follow-up of enrolled participants was performed by sending reminders of the sampling days and sending weekly emails/text messages to remind participants that they should consult the site clinics/study personnel in case of presenting symptoms related to dengue.

### 2.4. Study Objectives and Endpoints

The primary objective of this study was to determine the incidence of DENV infection, both symptomatic and asymptomatic infections, among students entering universities and technical schools in Medellín, their families, and employees of partner companies associated with PECET. Participants should not have had anti-DENV antibodies at the time of enrollment. As the aim was to establish the potential of this population as a proxy for travelers in a future dengue antiviral prophylaxis study, the participants should not have had anti-DENV antibodies at the time of enrollment.

Laboratory-confirmed DENV seroconversion was defined as having at least one positive anti-DENV IgG result at a post-baseline serosurvey visit during this study.

Symptomatic participants, experiencing a febrile illness episode, were defined to have a laboratory-confirmed acute DENV infection if DENV NS1 and/or DENV RNA were positive at the time of first evaluation (Day 0) in the febrile illness episode.

### 2.5. Study Assessments and Procedures

At screening, a serum sample was collected for anti-DENV IgG testing (using Dengue IgG Indirect ELISA, Panbio, Brisbane, Australia). The screening sample of enrolled participants was used as the baseline sample for further analyses. During the scheduled serosurvey visits, blood samples were collected for anti-DENV IgG testing (using Panbio Dengue IgG Indirect ELISA). The test result was considered positive if the PanBio Units calculated from the absorbance of the sample were >11, equivocal for PanBio Units ≥ 9 and ≤11, and negative for PanBio Units < 9, according to the manufacturer’s instructions. In case of a positive or equivocal anti-DENV IgG result, anti-DENV IgM (using Dengue IgM Capture ELISA, Panbio, Brisbane, Australia) was measured at all serosurvey visits for the respective participants. In addition, possible cross-reactivity of the Panbio Dengue IgG Indirect ELISA with anti-Zika virus (ZIKV) antibodies was assessed for all participants with at least one positive or equivocal anti-DENV IgG result by anti-ZIKV IgG testing (using the anti-ZIKV IgG ELISA, EuroImmun, Lübeck, Germany) of the baseline sample, as well as the last positive sample or the sample with the highest anti-DENV IgG antibody titer. The anti-ZIKV IgG ELISA test result was considered positive if the ratio of the extinction of the sample to the calibrator was ≥1.1, borderline if ≥0.8 to <1.1, and negative if <0.8.

During the febrile illness visits for participants with fever (≥38 °C) or a history of fever in the preceding 7 days or less, a medical examination was performed, standard of care for evaluation of suspected dengue was provided, and participants were to be hospitalized, if needed. A blood sample was collected, and a DENV NS1 rapid test (Dengue NS1 Detect Rapid Test, InBios International Inc., Seattle, WA, USA) was performed. Participants were subsequently followed up for 14 days (site visit) or 21 days (home visit), with blood sample collection on Day 14 or Day 21. Anti-DENV IgM was tested on the 14 (or Day 21) sample. If DENV NS1 and/or anti-DENV IgM ELISA were positive for DENV infection at any of the assessed time points, a qualitative DENV/chikungunya virus (CHIKV)/ZIKV real-time RT-PCR (Trioplex real-time RT-PCR assay) was performed on the Day 0 sample. Anti-ZIKV and anti-CHIKV IgM ELISA (anti-CHIKV IgM ELISA, EuroImmun, Lübeck, Germany and anti-Zika virus IgM ELISA, EuroImmun, Lübeck, Germany) were performed on all collected samples during the febrile illness episode. For both assays, a test result was considered positive if the ratio of the extinction of the sample to the calibrator was ≥1.1, borderline if ≥0.8 to <1.1, and negative if <0.8.

### 2.6. Statistical Analysis

The analysis of screening anti-DENV IgG data to identify DENV seronegative participants was performed for all participants who signed informed consent and for whom a screening blood serum sample was available. The analyses of primary DENV infection (DENV seroconversion and acute DENV infection) were conducted for all participants who signed informed consent, had a screening blood sample negative for anti-DENV IgG, and were enrolled in this study.

### 2.7. Sample Size

No formal sample size calculation was performed. Up to 2150 participants were planned to be recruited in Cohorts I and II: 1075 for Cohort I (2018–2020) and 1075 for Cohort II (2019–2021). For Cohort III, the aim was to enroll 1262 participants among university students, relatives, and employees of partner companies associated with PECET.

## 3. Results

### 3.1. Screened Participants

#### 3.1.1. Disposition

Of the 4885 participants screened (1803 in Cohort I, 1797 in Cohort II, and 1285 in Cohort III), 4881 participants had serology data available at screening.

#### 3.1.2. Demographic and Baseline Characteristics of Screened Participants

The median age of screened participants was 19 years (range 16 to 60 years) and 20 years (range from 16 to 64 years) for Cohorts I and II, respectively, versus 30 years (range 17 to 77 years) and 25 years (range 16 to 76 years) for Cohorts IIIA and III B, respectively (Table 1). All participants screened for Cohorts I and II were below 65 years of age, while 5 (2.5%) and 19 (1.7%) participants in Cohort IIIA and B, respectively, were above 65 years of age. Almost all participants (99.9%) in Cohorts I and II were recruited from educational institutions, while these represented 21.8% of recruitment in Cohort IIIA and 88.5% of recruitment in Cohort IIIB.

Anti-DENV serology data at screening were available for 4881/4885 screened participants. Overall, 1873/4881 (38.4%) screened participants with serology data available had anti-DENV IgG antibodies at screening, and 3008/4881 (61.6%) were seronegative. Among participants screened for Cohorts I and II, anti-DENV IgG antibodies were detected in 1423/3596 (39.6%) participants and not in the remaining 2173/3596 (60.4%) participants. In Cohort III, 450/1285 (35.0%) screened participants were positive for anti-DENV IgG antibodies, and 835/1285 (65.0%) were seronegative (Table 2). A higher DENV seroprevalence was found among participants ≥65 years (overall DENV seroprevalence of 62.5% [15/24]) and for participants of Black or African American race (overall DENV seroprevalence of 84.4% [103/122]).

### 3.2. Enrolled Participants

#### 3.2.1. Disposition

Of the 4881 participants with serology data at baseline, 3008 (61.6%) were dengue seronegative and were enrolled in this study, of which 2263 (75.2%) enrolled participants completed this study. The reasons for premature study termination were lost to follow-up for 732 (24.3%) participants and study withdrawal by the participant for 13 (0.4%) participants.

#### 3.2.2. Demographic and Baseline Characteristics of Enrolled Participants

Overall, a higher proportion of enrolled participants were female (64.2%), and the majority were of mixed race (69.9%) (Table 3). The median age was 19 years (range 16 to 60 years) and 20 years (range 16 to 39 years) for Cohorts I and II, respectively, versus 28 years (range 17 to 77 years) and 23 years (range 16 to 72 years) for Cohorts IIIA and B, respectively.

#### 3.2.3. Evaluation of DENV Seroconversion in Enrolled Participants

Over the study period, paired serosurvey visit data, i.e., from the baseline visit and at least 1 post-baseline serosurvey visit, were available for a total of 2644 participants across the three cohorts. The overall laboratory-confirmed DENV seroconversion rate in this study was 2.0% (52/2644) (Table 4). On an annual basis, the DENV seroconversion rate was the highest in 2022 (2.0% [15/737 participants with paired serosurvey visit data for 2022]), while rates were lower for 2020 and 2021 (0.5% [8/1641] and 0.4% [3/755], respectively).

Anti-DENV IgM data from the serosurvey visits could be generated for 45/52 participants with anti-DENV IgG seroconversion, while for the remaining 7 participants, insufficient sample volume was available. For 6 participants, anti-DENV IgM antibodies were detected at the first serosurvey visit, positive for anti-DENV IgG antibodies, suggesting that these participants had potentially had DENV infection at the time of the respective serosurvey visit or a recent prior DENV infection.

Cross-reactivity with IgG antibodies against ZIKV, a related flavivirus, was evaluated for 44 out of the 52 participants with DENV seroconversion for whom sufficient sample volume was available. Four participants had anti-ZIKV IgG antibodies already at the baseline visit, when anti-DENV IgG was not detected. For 4 other participants with DENV seroconversion, anti-ZIKV IgG antibodies became positive at 1 or 2 follow-up serosurvey visits, while not detected at baseline. For 3 out of these 4 participants, anti-ZIKV IgG and anti-DENV IgG antibodies were detected at the same visit.

#### 3.2.4. Clinically-Confirmed DENV Infections

Among the 3008 participants enrolled, 19 participants (<1%) presented at the site with a febrile illness episode (with fever ≥38 °C or history of fever in the preceding 7 days or less). Acute DENV infection was not laboratory-confirmed in any of the 19 symptomatic participants during their febrile illness episode (Table 5). The DENV NS1 rapid test performed on Day 0 of presentation to the clinical site was negative for all 19 participants. For 3 out of the 19 participants, anti-DENV IgM antibodies were detected on Day 14. For these 3 participants, DENV, ZIKV, and CHIKV RNA levels were undetectable in a Trioplex DENV/ZIKV/CHIKV RT-PCR performed on their Day 0 samples. Furthermore, in each of these 3 participants, anti-DENV IgG antibodies remained negative during all serosurvey visits following the febrile illness episode. Anti-DENV IgG also remained negative at subsequent serosurvey visits for all other symptomatic participants. Note that for four of these, the last serosurvey visit coincided with the febrile illness event (n = 3) or was before the event (n = 1).

Fourteen out of the 19 symptomatic participants experienced fever in the last 24 h prior to presentation. For 5 of these 14 participants, the total duration of fever prior to presentation at the site was less than 24 h, and longer than 24 h for 9 of these 14 participants, with a median of 3 days. All 19 participants experienced fever and at least two other symptoms meeting the World Health Organization (WHO) clinical diagnosis criteria for a probable clinical dengue case [21]. Other symptoms than fever reported at presentation at the site included headache, chills, feeling tired and loss of appetite in 18 participants; eye symptoms, muscle aches and feeling feverish in 17 participants; bone pain and nausea or vomiting in 13 participants; abdominal pain in 8 participants; diarrhea in 4 participants and rash or itchy skin in 2 participants.

Serology was performed to evaluate the presence of anti-ZIKV or anti-CHIKV IgM antibodies on Day 0 or on Day 14 (or 21) to evaluate a potential current ZIKV or CHIKV infection. None of the symptomatic participants had detectable anti-ZIKV IgM antibodies. In one participant, anti-CHIKV IgM antibodies were detected on Day 0, while anti-DENV IgM was negative for this participant.

## 4. Discussion

With the rise in global travel, DENV infections have become a significant public health concern, also for those visiting endemic regions. Evaluation of antiviral or vaccination strategies for the prevention of DENV infections in a traveler population would require extensive and complex studies. This prospective study was designed to assess the incidence of symptomatic and asymptomatic DENV infections in a dengue-naïve population residing in a dengue-endemic region in Medellín in the Aburrá Valley of Antioquia, Colombia, as a proxy for a traveler’s population. Therefore, the study population was recruited among a population of young participants, highly mobile, living mostly on the outskirts of the city of Medellín, moving to the city of Medellín to complete their studies (technical school or university), and who could be followed up prospectively within the PECET network. Furthermore, to function as a proxy of a traveler’s population, being dengue naïve is a key factor, as travelers originate in non-dengue-endemic areas and travel to dengue-endemic regions. While this approach could also be applicable to other regions globally, the altitude variations within the Medellín region offer a distinctive opportunity to identify dengue naïve individuals transitioning to an endemic area. The study seroprevalence indicated that 60% of the screened participants for Cohorts I and II were dengue naïve. For Cohort III, the proportion of dengue-naïve participants of 65% was similar to Cohort I and II even though the median age in this cohort (30 years in Cohort IIIA and 25 years in Cohort IIIB) was higher than that of Cohorts I and II (19 years in Cohort I and 20 years in Cohort II). Studies have shown that the presence of anti-DENV IgG antibodies in exposed populations is associated with increasing age [22].

Finding a dengue-naïve population or a cohort within a population in a dengue-endemic region is not an easy task. In serosurvey studies conducted in Colombia, anti-DENV antibody prevalence of 30% to 70% was described [4,23,24]. In a serosurvey study conducted between 2011 and 2014 in the Santa Cruz Comuna of Medellín, performed shortly after the 2010 dengue outbreak, an overall DENV seroprevalence of 61% was reported, with significantly higher dengue seroprevalence among older age groups [4].

Dengue virus is the leading cause of fever in travelers from non-endemic regions traveling to dengue-endemic areas. Based on prospective studies, the incidence rate for symptomatic DENV infections among travelers to the endemic areas of Asia, Africa, and the Americas was estimated at 6 per 1000 person-months of travel [25]. Based on serological data, including asymptomatic infections, an incidence rate of 47 per 1000 person-months of travel was estimated for Dutch travelers to Suriname [26]. Most dengue cases among travelers are primary infections, and severe dengue disease is rare. Over the study period from 2018 to 2022, the incidence of DENV seroconversion among enrolled participants was 2.0% (52/2644), and no laboratory-confirmed acute symptomatic dengue cases were detected. During this time, the Aburrá Valley area did not experience any dengue epidemics and the numbers of annual reported dengue cases, including probable and confirmed cases, for the city of Medellín were substantially lower than in the 2016 epidemic year (1175 in 2018, 1244 in 2019, 611 in 2020, 240 in 2021 and 228 in 2022 compared to 17,394 in 2016) [27].

DENV seroconversion was defined as having at least one positive anti-DENV IgG result at a post-baseline serosurvey visit during this study. The co-circulation of ZIKV, a related flavivirus transmitted by the same *Aedes* mosquito vector, may, however, lead to cross-reactivity between anti-ZIKV and anti-DENV antibodies in serology testing. This was investigated by determination of anti-ZIKV IgG for those participants with a positive anti-DENV IgG result. Potential cross-reactivity may have been present for 8 participants with DENV seroconversion, as anti-ZIKV IgG was positive at baseline or became positive at a post-baseline serosurvey visit.

This study has limitations, the first being the timing of a dengue epidemic. The presence of the mosquito vector is driven by climatic phenomena typical of tropical regions, such as the El Niño phenomenon [28], which, during the time of this study, did not affect Colombia [29]. A higher incidence of DENV seroconversion in the city of Medellín, with seroconversion detected in 122 out of 1002 dengue-naïve participants, was reported in the serosurvey study conducted by Carabali and colleagues between 2011 and 2014, which included the 2013 dengue outbreak in Colombia [4]. The most recent dengue epidemic in Medellín took place in the year 2016, with 17,394 cases reported in Medellín [27], and was preceded by one of the strongest El Niño phenomena on record that was observed from the second half of 2015 until May 2016 [29]. During the first half of 2024, a substantial increase in reported dengue cases was observed in Medellín [27]. Colombia officially declared the presence of the El Niño phenomenon in November 2023, and it may be that the El Niño-driven dengue epidemic in Colombia was manifesting itself in the Aburrá area [30,31]. For a vaccine or an antiviral study using the same approach as this study, including a proxy for travelers, the cyclical pattern of El Niño for Medellín will have to be taken into account. For Medellín, the El Niño effect may have started in the year 2024, a year outside of the study period [31].

Secondly, during the conduct of this study, the World Mosquito Program (WMP), a biological control strategy releasing *Wolbachia* mosquito populations to lessen virus transmission, was implemented in Medellín from 2018 to 2021 [32]. The rate of dengue case notifications in Medellín declined by 95% after *Wolbachia* releases compared to the pre-intervention period [32]. A case-controlled study performed in Medellín showed that laboratory-confirmed dengue incidence was significantly lower in three neighborhoods following the release of the wMel strain of *Wolbachia* compared to three adjacent untreated neighborhoods [32]. However, it is important to consider that only 115 (3.8%) participants were included in this study who lived in Manrique, Aranjuez, or Santa Cruz, the three neighborhoods where the *Wolbachia* strategy was implemented, and thus, a relevant effect on the incidence in this study can be excluded.

Another limitation of this study concerned the concurrence of study Cohorts II and III with the COVID-19 pandemic. The associated lockdown and isolation measures may have deterred participants who experienced febrile illness from site or home visits and thereby may have limited the detection of symptomatic dengue cases. Furthermore, despite participants receiving training for dengue awareness and identification of dengue symptoms, self-reporting of febrile illness may also have led to underreporting of symptomatic cases. Finally, an important consideration in defining laboratory-confirmed acute DENV infections is the characterization of symptoms and adequate timing of diagnostic sample collection. The time between the onset of symptoms and sample collection may impact the detection of DENV NS1 antigen or DENV RNA, as these are generally only detected during the early phase of the disease. For the 14 participants with fever in the past 24 h, the median duration of fever was 3 days, indicating that the time between the onset of symptoms and sample collection was 3 days or longer for at least half of them. Three symptomatic participants had anti-DENV IgM antibodies 14 days after presentation to the site with fever. However, for none of these 3 participants, DENV RNA or DENV NS1 could be detected in the samples collected at presentation to the site, and anti-DENV IgG remained negative at all subsequent serosurvey visits for each of the 3 participants, and these were therefore, although suggestive of DENV infection, not considered as laboratory-confirmed dengue cases. Anti-DENV IgG also remained negative for all other symptomatic participants for whom subsequent serosurvey visits were completed. Nevertheless, based on clinical symptoms, all 19 participants with a fever episode (ongoing or history of fever in the preceding 7 days or less) could be classified as probable clinical dengue cases, as all had at least two additional symptoms meeting the WHO dengue clinical diagnosis criteria [21].

## 5. Conclusions

The current study demonstrated the feasibility of identifying a substantial dengue-naïve cohort of people willing to participate and complete a dengue study among a population of predominantly students living in the metropolitan area of Medellín. This cohort can, in fact, serve as a proxy for a traveler’s population. The learnings from this study are intended to support the design and sample size determination of future dengue antiviral studies in a pre-exposure study design, and indeed, the high proportion of dengue-naïve individuals can support such a study. However, timing is of the essence, and the 2% seroconversion implies a larger sample size that can make this study expensive. Timing can benefit from a better understanding of the El Niño effect/presence in the Aburrá Valley, and/or timing such a study following an El Niño-influenced season could also help find a higher dengue transmission. Dengue vaccine studies that aim to understand the vaccination impact on the prevention of dengue infection could also benefit from this type of cohort.

## Figures and Tables

**Table 1:** Summary of demographics and characteristics of screened participants.

	Cohort				Total
	I	II	IIIA	IIIB	
Screened participants	1803	1797	197	1088	4885
Age, [years]					
N	1800	1797	197	1088	4882
Mean (SD)	20.36 (3.594)	20.41 (3.372)	33.54 (13.259)	29.90 (13.155)	23.04 (8.642)
Median	19.00	20.00	30.00	25.00	20.00
Range	(16.0; 60.0)	(16.0; 64.0)	(17.0; 77.0)	(16.0; 76.0)	(16.0; 77.0)
<65	1800 (100%)	1797 (100%)	192 (97.5%)	1069 (98.3%)	4858 (99.5%)
≥65	0	0	5 (2.5%)	19 (1.7%)	24 (0.5%)
Unknown	3	0	0	0	3
Sex, n (%)					
N	1803	1797	197	1088	4885
Female	1092 (60.6%)	1179 (65.6%)	99 (50.3%)	709 (65.2%)	3079 (63.0%)
Male	711 (39.4%)	618 (34.4%)	98 (49.7%)	379 (34.8%)	1806 (37.0%)
Race, n (%)					
N	1742	1796	197	1084	4819
Mixed White/American Indian	1666 (95.6%)	1695 (94.4%)	0	0	3361 (69.7%)
Black or African American	42 (2.4%)	73 (4.1%)	1 (0.5%)	6 (0.6%)	122 (2.5%)
White	33 (1.9%)	27 (1.5%)	196 (99.5%)	1077 (99.4%)	1333 (27.7%)
Multiple	1 (0.1%)	1 (0.1%)	0	1 (0.1%)	3 (0.1%)
Unknown	61	1	0	4	66
Ethnicity, n (%)					
N	1799	1794	96	894	4583
Hispanic or Latino	1240 (68.9%)	1794 (100%)	96 (100%)	893 (99.9%)	4023 (87.8%)
Not Hispanic or Latino	559 (31.1%)	0	0	1 (0.1%)	560 (12.2%)
Unknown	4	3	101	194	302
Workplace during daytime, n (%)					
N	1803	1797	197	1088	4885
Universidad de Antioquia	1801 (99.9%)	979 (54.5%)	1 (0.5%)	419 (38.5%)	3200 (65.5%)
Universidad Cooperativa de Colombia	0	427 (23.8%)	0	17 (1.6%)	444 (9.1%)
Corporación Universitaria Remington	0	389 (21.6%)	0	20 (1.8%)	409 (8.4%)
Colegio Mayor de Antioquia (Colmayor)	0	0	28 (14.2%)	234 (21.5%)	262 (5.4%)
Instituto Tecnológico Metropolitano (ITM)	0	0	8 (4.1%)	96 (8.8%)	104 (2.1%)
Fundación Universitaria San Martín	0	0	0	92 (8.5%)	92 (1.9%)
Universitaria Pascual Bravo	0	0	2 (1.0%)	54 (5.0%)	56 (1.1%)
Corporación Universitaria de Sabaneta (Unisabaneta)	0	0	4 (2.0%)	31 (2.8%)	35 (0.7%)
Partner Company Arary S.A.S.	0	0	65 (33.0%)	0	65 (1.3%)
Partner Company EyD Estructuras y Desarrollos	0	0	40 (20.3%)	0	40 (0.8%)
Other ^a^	2 (0.1%)	2 (0.1%)	49 (24.9%)	125 (11.5%)	178 (3.6%)

N, total number of participants per group; n (%), number (percentage) of participants in the specified category; SD, standard deviation. ^a^ Other: locations with less than 30 screened subjects, including family members of students and students/workers at other universities or companies.

**Table 2:** Screened participants: summary of dengue seroprevalence.

	Cohort				Total
	I	II	IIIA	IIIB	
Screened participants	1803	1797	197	1088	4885
Overall, n (%)					
N	1799	1797	197	1088	4881
Negative	1122 (62.4%)	1051 (58.5%)	111 (56.3%)	724 (66.5%)	3008 (61.6%)
Positive	677 (37.6%)	746 (41.5%)	86 (43.7%)	364 (33.5%)	1873 (38.4%)
Sex, n (%)					
Female					
N	1089	1179	99	709	3076
Negative	686 (63.0%)	707 (60.0%)	58 (58.6%)	481 (67.8%)	1932 (62.8%)
Positive	403 (37.0%)	472 (40.0%)	41 (41.4%)	228 (32.2%)	1144 (37.2%)
Male					
N	710	618	98	379	1805
Negative	436 (61.4%)	344 (55.7%)	53 (54.1%)	243 (64.1%)	1076 (59.6%)
Positive	274 (38.6%)	274 (44.3%)	45 (45.9%)	136 (35.9%)	729 (40.4%)
Age, n (%)					
<65 years					
N	1799	1797	192	1069	4857
Negative	1122 (62.4%)	1051 (58.5%)	109 (56.8%)	717 (67.1%)	2999 (61.7%)
Positive	677 (37.6%)	746 (41.5%)	83 (43.2%)	352 (32.9%)	1858 (38.3%)
≥65 years					
N	0	0	5	19	24
Negative	0	0	2 (40.0%)	7 (36.8%)	9 (37.5%)
Positive	0	0	3 (60.0%)	12 (63.2%)	15 (62.5%)
Race, n (%)					
Mixed White/American Indian					
N	1665	1695	0	0	3360
Negative	1047 (62.9%)	1023 (60.4%)	0	0	2070 (61.6%)
Positive	618 (37.1%)	672 (39.6%)	0	0	1290 (38.4%)
Black or African American					
N	42	73	1	6	122
Negative	11 (26.2%)	6 (8.2%)	0	2 (33.3%)	19 (15.6%)
Positive	31 (73.8%)	67 (91.8%)	1 (100%)	4 (66.7%)	103 (84.4%)
White					
N	33	27	196	1077	1333
Negative	23 (69.7%)	21 (77.8%)	111 (56.6%)	717 (66.6%)	872 (65.4%)
Positive	10 (30.3%)	6 (22.2%)	85 (43.4%)	360 (33.4%)	461 (34.6%)
Multiple					
N	1	1	0	1	3
Negative	1 (100%)	0	0	1 (100%)	2 (66.7%)
Positive	0	1 (100%)	0	0	1 (33.3%)
Ethnicity, n (%)					
Hispanic or Latino					
N	1239	1794	96	893	4022
Negative	781 (63.0%)	1050 (58.5%)	54 (56.3%)	596 (66.7%)	2481 (61.7%)
Positive	458 (37.0%)	744 (41.5%)	42 (43.8%)	297 (33.3%)	1541 (38.3%)
Not Hispanic or Latino					
N	559	0	0	1	560
Negative	341 (61.0%)	0	0	0	341 (60.9%)
Positive	218 (39.0%)	0	0	1 (100%)	219 (39.1%)

N, total number of participants per group; n (%), number (percentage) of participants in the specified category.

**Table 3:** Enrolled participants: summary of demographics and baseline characteristics.

	Cohort				Total
	I	II	IIIA	IIIB	
Enrolled Participants	1122	1051	111	724	3008
Age, [years]					
N	1122	1051	111	724	3008
Mean (SD)	20.17 (3.393)	20.31 (2.903)	32.15 (13.307)	27.81 (11.588)	22.50 (7.741)
Median	19.00	20.00	28.00	23.00	20.00
Range	(16.0; 60.0)	(16.0; 39.0)	(17.0; 77.0)	(16.0; 72.0)	(16.0; 77.0)
<65	1122 (100%)	1051 (100%)	109 (98.2%)	717 (99.0%)	2999 (99.7%)
≥65	0	0	2 (1.8%)	7 (1.0%)	9 (0.3%)
Sex, n (%)					
N	1122	1051	111	724	3008
Female	686 (61.1%)	707 (67.3%)	58 (52.3%)	481 (66.4%)	1932 (64.2%)
Male	436 (38.9%)	344 (32.7%)	53 (47.7%)	243 (33.6%)	1076 (35.8%)
Race, n (%)					
N	1082	1050	111	720	2963
Mixed race	1047 (96.8%)	1023 (97.4%)	0	0	2070 (69.9%)
Black or African American	11 (1.0%)	6 (0.6%)	0	2 (0.3%)	19 (0.6%)
White	23 (2.1%)	21 (2.0%)	111 (100%)	717 (99.6%)	872 (29.4%)
Multiple	1 (0.1%)	0	0	1 (0.1%)	2 (0.1%)
Unknown	40	1	0	4	45
Ethnicity, n (%)					
N	1122	1050	54	596	2822
Hispanic or Latino	781 (69.6%)	1050 (100%)	54 (100%)	596 (100%)	2481 (87.9%)
Not Hispanic or Latino	341 (30.4%)	0	0	0	341 (12.1%)
Unknown	0	1	57	128	186
Workplace during daytime					
N	1122	1051	111	724	3008
Universidad de Antioquia	1121 (99.9%)	609 (57.9%)	1 (0.9%)	297 (41.0%)	2028 (67.4%)
Universidad Cooperativa de Colombia	0	247 (23.5%)	0	16 (2.2%)	263 (8.7%)
Corporación Universitaria Remington	0	193 (18.4%)	0	19 (2.6%)	212 (7.0%)
Colegio Mayor de Antioquia (Colmayor)	0	0	18 (16.2%)	155 (21.4%)	173 (5.8%)
Instituto Tecnológico Metropolitano (ITM)	0	0	5 (4.5%)	56 (7.7%)	61 (2.0%)
Fundación Universitaria San Martín	0	0	0	56 (7.7%)	56 (1.9%)
Universitaria Pascual Bravo	0	0	1 (0.9%)	32 (4.4%)	33 (1.1%)
Corporación Universitaria de Sabaneta (Unisabaneta)	0	0	3 (2.7%)	21 (2.9%)	24 (0.8%)
Partner Company Arary S.A.S.	0	0	34 (30.6%)	0	34 (1.1%)
Partner Company EyD Estructuras y Desarrollos	0	0	18 (16.2%)	0	18 (0.6%)
Other ^a^	1 (0.1%)	2 (0.2%)	31 (27.9%)	72 (9.9%)	106 (3.5%)
Previously vaccinated for Yellow Fever, n (%)					
Yes	298 (26.6%)	479 (45.6%)	32 (28.8%)	302 (41.7%)	1111 (36.9%)
No	824 (73.4%)	572 (54.4%)	79 (71.2%)	422 (58.3%)	1897 (63.1%)

N, total number of participants per group; n (%), number (percentage) of participants in the specified category; SD, standard deviation. ^a^ Other: locations with less than 30 screened subjects, including family members of students and students/workers at other universities or companies.

**Table 4:** Incidence of DENV seroconversion in enrolled participants.

	Participants with Paired Serosurveys, n	Laboratory-Confirmed n (%)	Laboratory-Negative n (%)
2018 Semester 2	863	11 (1.3%)	852 (98.7%)
2018 Total	863	11 (1.3%)	852 (98.7%)
2019 Semester 1	881	3 (0.3%)	878 (99.7%)
2019 Semester 2	1675	12 (0.7%)	1663 (99.3%)
2019 Total	1755	15 (0.9%)	1740 (99.1%)
2020 Semester 1	1576	8 (0.5%)	1568 (99.5%)
2020 Semester 2	594	0 (0.0%)	594 (100%)
2020 Total	1641	8 (0.5%)	1633 (99.5%)
2021 Semester 1	645	2 (0.3%)	643 (99.7%)
2021 Semester 2	110	1 (0.9%)	109 (99.1%)
2021 Total	755	3 (0.4%)	752 (99.6%)
2022 Semester 1	698	13 (1.9%)	685 (98.1%)
2022 Semester 2	619	2 (0.3%)	617 (99.7%)
2022 Total	737	15 (2.0%)	722 (98.0%)
Total	2644	52 (2.0%)	2592 (98.0%)

Paired serosurvey is defined as the availability of anti-DENV IgG data at baseline and at least one anti-DENV IgG result in the specific time interval. Laboratory-confirmed DENV infection is defined as at least one positive post-baseline result for anti-DENV IgG.

**Table 5:** Febrile illness episodes for enrolled participants.

	Total (n = 3008)
Number of participants with a febrile illness episode	19
Positive DENV NS1 rapid test, n	0
Positive for anti-DENV IgM antibodies (Day 14), n	3
Positive for DENV RNA (RT-PCR ^a^), n	0
Positive for ZIKV RNA (RT-PCR ^a^), n	0
Positive for CHIKV RNA (RT-PCR ^a^), n	0

CHIKV, chikungunya virus; DENV, dengue virus; Ig, immunoglobulin; n, number of participants; NS1, nonstructural protein 1; RT-PCR, reverse transcription polymerase chain reaction; ZIKV, Zika virus. ^a^ Trioplex real-time RT-PCR (DENV, ZIKV, CHIKV) was only performed for participants with positive anti-DENV IgM.

## Data Availability

All available data are presented in the article.

## Abbreviations

The following abbreviations are used in this manuscript:

CHIKV	Chikungunya virus
COVID-19	coronavirus disease-2019
DENV	dengue virus
ELISA	enzyme-linked immunosorbent assay
Ig	immunoglobulin
ITM	Instituto Tecnológico Metropolitano
NS1	nonstructural protein 1
PECET	Programa de Estudio y Control de Enfermedades Tropicales
SD	standard deviation
WHO	World Health Organization
WMP	World Mosquito Program
ZIKV	Zika virus
